# Safeguarding athletes and anti-doping: applying theories of vulnerability

**DOI:** 10.3389/fspor.2025.1512541

**Published:** 2025-05-22

**Authors:** Angela J. Schneider, Nelson Morales Páez, Yaneí Lezama Ramírez, Loughran Butcher

**Affiliations:** ^1^ International Centre for Olympic Studies, Western University, London, ON, Canada; ^2^ Faculty of Health Sciences, School of Kinesiology, Western University, London, ON, Canada; ^3^ Ethics and Public Affairs, Carleton University, Ottawa, ON, Canada

**Keywords:** safeguarding, athletes, anti-doping, theories of vulnerability, feminist theory

## Abstract

‘Safeguarding' in sport has been a fast-growing movement, particularly in the past decade, which currently encompasses a field of study and a policy development strategy. Although it is greatly needed in all sectors of sport, the concept and application of safeguarding to anti-doping has been underexplored and under theorized. In this article, utilizing the method of critical philosophical and ethical analysis, we attempt to provide evidence regarding why the intersection between safeguarding and anti-doping is very important and requires critical analysis; moreover, we suggest that feminist bioethics reflections on vulnerability can offer unique insights into key issues related to safeguarding in sport, such as the autonomy of athletes and the concept of ‘protected persons' and, most pertinent to this research, to the concepts of athlete vulnerability and anti-doping in sport. We explore the concept of vulnerability within the context of doping and anti-doping. We examine the etymology of vulnerability, discuss contemporary theories, particularly those based on biomedical ethics and feminist theories, and apply these ideas to context of anti-doping in sport. We also address the concept of safeguarding in sport, focusing on its current definitions and applications and identify gaps in the literature where doping is not yet considered a safeguarding issue. Through discussion, we link the concept of vulnerability with safeguarding by analyzing specific anti-doping cases where athlete vulnerability can, and has, resulted in significant harm to athletes’ integrity and wellbeing. These cases are from situations with minors, and they serve as a platform to put forward an integrated approach for policy development that draws on feminist theories of vulnerability, safeguarding, and biomedical ethics principles. In the results presented in the summary and conclusions, we discuss how insights from feminist theories and biomedical ethics can contribute to more effective safeguarding policies, emphasizing the importance of prevention and education rather than just the current kind of safeguarding measures that are predominantly punitive. We conclude by advocating for the urgent implementation of comprehensive safeguarding measures that address the vulnerabilities associated with anti-doping amongst athletes at all levels. This approach should prioritize prevention, fostering a balanced system that emphasizes education and awareness; where education is not just solely related to individual agency and educating athletes, but also about educating all of the anti-doping movement stakeholders to understand the particular role they play in the circumstances that increase vulnerability so that the risks can be mitigated structurally as well. To achieve this end, it is essential to develop educational programs that not only inform athletes about the risks and consequences of doping, but also empowers them with knowledge about their rights and responsibilities within the sporting community and the responsibilities of other stakeholders within the anti-doing movement. These safeguarding programs should be designed not only to promote resilience against external pressures, but, in particular, to reduce vulnerability that is created structurally more broadly speaking in the anti-doping context.

## 1 Introduction

Doping remains a pervasive issue in elite sport, despite decades of global anti-doping efforts that rely on deterrence, sanctions, and education. Much of the existing research on doping focuses on individual factors, such as athletes' values, ethical decision-making, and moral engagement with principles like fair play and clean sport (1–3). While these factors are undoubtedly important, this narrow focus overlooks the broader structural and situational factors that contribute to athletes' vulnerability to doping. For example, high-pressure environments, power imbalances, and systemic failures in safeguarding policies can exacerbate athletes' susceptibility to harm, including the use of performance-enhancing substances.

In this paper we argue that safeguarding in sport, a growing movement aimed at protecting athletes from harm, must be expanded to address: (i) underpinning theories of vulnerability; (ii) the vulnerabilities that predispose athletes to doping; and (iii) the ethical, philosophical and structural dimensions of vulnerability in the anti-doping domain. Current safeguarding literature often focuses on issues like harassment, abuse, and misconduct, but it rarely considers doping as a safeguarding issue. Similarly, anti-doping policies tend to emphasize punitive measures rather than addressing the root causes of athletes' vulnerability. Building on perspectives from feminist bioethics and theories of vulnerability, we seek to bridge these gaps and propose a more comprehensive approach to safeguarding in the context of anti-doping. In order to develop a comprehensive ethical framework for safeguarding athletes, we also incorporate psychological research that empirically documents how vulnerabilities manifest in real-world anti-doping contexts. These psychological perspectives provide crucial insights into athletes' situational and systemic pressures, thereby complementing and operationalizing the relational dimension of vulnerability theorized in normative ethics. By integrating these perspectives, we aim to ensure that ethical reflections are informed by lived experience and can guide more grounded policy recommendations. This interdisciplinary strategy, blending normative theory with psychological evidence, is essential to understanding vulnerability in both its conceptual and applied dimensions.

First, we explore how concepts of vulnerability, both ontological (universal) and relational (context-specific), can deepen our understanding of athletes' vulnerabilities in the anti-doping context; and second, we demonstrate the need for safeguarding policies to be reimagined to address these vulnerabilities by identifying the ethical, philosophical and structural dimensions of vulnerability in the anti-doping domain and that the concept and application of safeguarding to anti-doping has been underexplored and under-theorized.

This paper is structured into four key sections, each building on the central theme of integrating safeguarding and vulnerability theories into anti-doping policies to better protect athletes. The first section, “Definitions and Theories of Vulnerability” provides a theoretical framework that introduces the concept of vulnerability, distinguishing between its two dimensions: ontological vulnerability, which refers to the universal susceptibility to harm inherent in the human condition, and relational vulnerability, which is context-specific and shaped by social, cultural, and environmental factors. We examine the etymology of vulnerability, discuss contemporary theories, particularly those based on biomedical ethics and feminist theories, and apply these ideas to the context of anti-doping. This theoretical foundation is critical for understanding how safeguarding can address these vulnerabilities and provides the conceptual basis for the main purpose and arguments in this paper.

The second section, “Definition of Safeguarding,” provides a review of the current state of safeguarding literature in sports, which primarily focuses on issues like harassment, abuse, and misconduct. We identify significant gaps in the literature, particularly the lack of attention to doping as a safeguarding issue. The section critiques the reactive nature of existing safeguarding policies, which often address harm after it has occurred, rather than proactively mitigating the structural and situational factors that increase athletes' vulnerability to doping. This critique sets the stage for integrating safeguarding into anti-doping efforts and highlights the need for a more comprehensive approach.

In Section 6, “Vulnerability in the Anti-Doping Context,” and Section 7, “Safeguarding, Vulnerability, and the anti-doping context,” we link the concept of vulnerability with safeguarding by analyzing specific anti-doping cases where athlete vulnerability can, and has, resulted in significant harm to athletes’ integrity and well-being. Drawing on the psychological literature, which has been dominant in providing insights into athletes’ behavior around doping, we complement our ethical analysis with empirical findings that help illuminate the structural and contextual factors contributing to such vulnerability. These cases serve as a basis to put forward an integrated approach for policy development that draws on feminist theories of vulnerability, safeguarding, and biomedical ethics principles. In particular, we identify the specific situation for minors in Section ’Safeguarding, Vulnerability, and minors in the anti-doping context’.

Finally, in Section 8, “Conclusions” we discuss how insights from the preceding sections can contribute to more effective safeguarding policies, emphasizing the importance of prevention and education rather than just the current model that is predominantly punitive. We conclude by advocating for the urgent implementation of comprehensive safeguarding measures that address the vulnerabilities associated with anti-doping amongst athletes at all levels but in particular for minors.

## 2 Methodology

Policy and decisions about safeguarding athletes in the anti-doping context in sport present value and ethics-based proposals: they are about what we ought to do, and therefore, they must be situated within a philosophical and ethical framework. The outcomes of empirical research are, at best, an improved understanding of factual matters, and so this “fact-value gap” can inform but cannot determine the best ethical policy and practice. The methodology utilized draws upon feminist epistemological and ethical standpoint theory (e.g., Sandra Harding (4) and Nancy Hartsock (5). This methodology assumes that knowledge claims are “always socially situated”, and also that those who are socially located as “insiders” have epistemological advantages in producing such knowledge. Standpoint theory further claims that in the process of knowledge production, the researcher's characteristics affect substantive and practical aspects. Standpoint epistemology enables scientists to draw upon their own experiences to determine “blind spots” in research processes, and this process results in an enhanced notion of objectivity where more positions are considered, and therefore, more thorough results are obtained (4). Transparency of the researcher's social location is important in preventing an “anonymous voice of authority,” and allows the reader to understand the researcher's position as a “real historical individual with concrete, specific desires and interests” (4). Within this research process, the primary investigator reflects on their “insider” positionality (which can be drawn from the identity as a former elite athlete and a former WADA director). By engaging with research reflexively, a better understanding of the athlete's experience in the anti-doping context is gained. These reflections not only inspired the questions asked but also allowed the researchers to pursue alternative perspectives to those discussed in current published research. This methodology enables the level of objectivity to be achieved, in part, through discussion and debate with diverse communities of sports ethicists. This process emphasizes the importance of socially situated knowledge, to advocate for athlete-centered safeguarding policies.

## 3 Relevant literature

There have been great inroads on the psychological research on vulnerability in the anti-doping context (6–9), an overview of much of it is given below. However, most of the current literature on sport safeguarding currently stems from a descriptive approach rather than a prescriptive one (i.e., descriptive “this is what is”, rather than prescriptive “this is the way it should be”), but what is required *is both*. Further, safeguarding literature on sport is often reactive as it focuses on identifying, mitigating, and punishing conduct that negatively impacts athletes' well-being, rather than focusing on the strategies necessary to prevent such conduct. In this safeguarding context, doping and anti-doping is usually not addressed. Neither are theories of what precisely “vulnerability” is, such as those presented below in this work. We explore concepts such as “inherent vulnerability” and “relational vulnerability” to better understand how these concepts may interplay within the World Anti-Doping Program as a whole, and then tie these concepts to Sport Safeguarding, both as a field of study and as a policy development initiative. By further developing theories of vulnerability within the context of sport, we offer new perspectives that can aid sport governing bodies and anti-doping organizations in creating more effective athlete-safeguarding policies. In turn, we propose a concept of safeguarding that is also functional for the development of anti-doping policies, primarily for the prevention of doping and the protection of athletes.

## 4 Definitions and theories of vulnerability

In general, we begin this section with the understanding that the conceptually underdetermined use and treatment of the concept of “vulnerability” (and or “susceptibility”) in anti-doping and safeguarding literature is somewhat tangled and ambiguous and is in need for a better theoretical foundation for its conceptualization, thus the rationale for the need for theorizing vulnerability and its relationship to safeguarding in the anti-doping context.

The concept of vulnerability has its etymological roots in the Latin word *vulnus*, meaning wound, injury, or harm, combined with the suffix *abilis*, which indicates possibility. In ancient Rome, the verb *vulnerare* referred to the act of injuring, while the term *vulnerable* (wound + possibility) denoted something capable of being harmed (10). In contemporary language, vulnerability retains a similar meaning, generally understood as susceptibility to being harmed. In more recent scholarly literature, vulnerability is often explored within the contexts of feminist philosophy and disability theory, where it is used to explain the susceptibility of certain individuals or groups to harm *vis-a-vis* other groups who do not seem to share such susceptibility (11–13). Within the philosophical debate about the meaning of and scope of vulnerability, one interpretation of the concept considers it a universal condition applicable to all individuals, given that, as human beings, we are embodied and have needs, and therefore we are inherently susceptible to harm, when our needs are unmet or when our bodies are injured (14). Since our needs are not merely physiological, but also affective, and sociopolitical (15, 16), our subsistence in need for the collective, challenges the traditional notion of human beings as entirely rational and self-sufficient agents, emphasizing instead our relational existence, which connects individuals with needs and interdependencies. It also supports a relational view of autonomy, which acknowledges that an individual's ability to make independent decisions is influenced by his or her relationships and social contexts surrounding them—an idea that will be further elaborated below, and which resonates with the findings from sociological (17, 18) and other empirical research (19) which introduce cultures and contexts as factors relevant to doping since at least 2008.

This notion of vulnerability as a universal quality is referred to as being “ontological” or “intrinsic” (11) and posits that the potential for suffering harm is a fundamental aspect of the human condition, arising from our embodied, socially affective, and sociopolitical existence (20, 21). As human beings we are constituted not only by our individual embodiment and need for care but also by our social interdependence with others and with institutions. In this way, humans are not only interconnected but also exposed to one another, rendering us susceptible to suffering due to dissatisfaction or the loss of these fundamental needs (14). We demonstrate in this article, that in the context of sport, this ontological vulnerability exists when speaking about inherent risks involved in training and competing at the elite level, as well as the susceptibility to injury and the pressures exerted by high-stakes environments.

Alternatively, vulnerability can be conceptualized more specifically to refer to individuals or groups with a heightened susceptibility to harm in comparison to others, due to reduced capacities for self-protection (20). In this more targeted context, vulnerability is often referred to as relational, and denotes a contingent condition, where specific factors such as social, physical, or environmental disadvantages place certain individuals or groups at greater risk of harm compared to the general population. This is the more prevalent understanding of vulnerability in everyday discourse as it is used to identify risks that are not equally prevalent for the entire human race but rather threatening to a greater extent specific sections of the population. At first glance, for example, many might not perceive someone like an Olympic athlete as vulnerable as they are physically outstanding; however, athletes are subject to numerous pressures, such as performance demands, institutional controls, and rigorous selection processes, all of which can importantly affect their well-being and integrity, thus constituting unique harm risks which may contribute to unique forms of vulnerability to which they are subject.

In biomedical ethics, this notion of relational vulnerability emphasizes the susceptibility of individuals or groups to harm, exploitation, or inherent injustices within the context of healthcare, biomedical research, and related domains (21, 22). This concept can also be extended to the realm of sport, and in particular, to doping and anti-doping, where athletes may be subject to similar harms, exploitation, or inherent injustices, for example, when pressured into using performance-enhancing drugs (clearly identified in the psychological research on vulnerability and doping reviewed below) or methods to remain competitive or when inadvertently becoming victims of contaminated substances. In these scenarios, the increased likelihood of these athletes engaging in doping practices from which they will derive harm, i.e., their increased vulnerability, may be shaped by various social, cultural, and environmental factors, such as the influence of coaches, financial pressures, lack of education, and the need for recognition.

While all human beings are inherently vulnerable, elite athletes can face unique threats that can also make them vulnerable to potential harms caused by arbitrariness and abuses of discretion involved in team selection processes and their ongoing participation in competitions requiring extensive monitoring of their lifestyles. During processes like these, athletes' vulnerability is further exacerbated by the significant power imbalances between them and sporting institutions, staff, and coaches. More recently, they are also exposed to more potential harm, and thus, vulnerability, through social media, causing increased public scrutiny, that can be very negative or even of a harassing nature, and the pressure to perform. If indeed processes such as those listed above do increase athletes' susceptibility to be harmed, from an ethical standpoint, sports authorities bear a moral responsibility to implement mechanisms that mitigate these potential harms. This can include addressing abuses of discretion occurring not only during the selection process but also in the continuous doping control and competition monitoring procedures. Such mechanisms might involve improving certainty and transparency in selection criteria, providing mental health support, ensuring fairness and transparency in anti-doping regulations, protocols, and practices like a clear sanction regime, consent obtention, testing protocols, fair results management, educated therapeutic use exemptions (TUE) applications, and protecting athletes from potentially coercive coaching environments as described below.

Relational vulnerability recognizes that while all humans are inherently vulnerable, certain individuals or groups experience heightened vulnerability due to their specific social, economic, or political contexts (20). This perspective emphasizes that vulnerability is not solely an intrinsic characteristic but is also shaped by external circumstances and relationships. We would further argue that elite athletes may be particularly vulnerable to exploitation or harm because of their dependence on institutional decisions, societal pressures, and strict regulatory controls. In the context of anti-doping, athletes are not just vulnerable to being coerced into doping, but also may be vulnerable to unfair treatment, no-fault ingestion, or false accusations, particularly when rules are unevenly implemented, testing protocols are inconsistently applied, or when athletes lack access to adequate legal representation.

By acknowledging both the ontological and relational dimensions of vulnerability, we can better understand how different individuals and groups experience harm in distinct ways, shaped by their particular contexts. Theories of vulnerability thus challenge broader ethical and philosophical debates, including the concept of autonomy in traditional Western liberal philosophy, by underscoring the limitations of viewing individuals as entirely independent agents (23). Vulnerability is not only a condition of human existence but also a universal quality for all human beings, by virtue of our embodied and social constitution (20, 22). In the context of sports, we argue that athletes' vulnerability should be emphasized in anti-doping (as is by much of the psychological research reviewed below), and further, that it should be tied to safeguarding discussions, as athletes are subject to intense scrutiny and regulation resulting in increased risks associated with their physical and social environments.

The ontological approach to vulnerability has raised critiques and important philosophical debate regarding the normative significance of the concept and its sufficiency to establish moral obligations (24). Critics like Sellman (25) argue that vulnerability could not possibly solely entail that all humans are inherently susceptible to harm, as this universal condition states the obvious and might not necessitate specific moral obligations. Sellman's work is relevant here because his specialization in health ethics, particularly in the ethics of nursing, brings the perspective of the ethics of care, which in line with feminist ethics, elevates *care* as a fundamental value that grounds moral responsibilities (i.e., duty of care). When vulnerability is instead used to identify a group as “more than ordinarily vulnerable,” an ethical responsibility arises to provide remedy by diminishing the potential harms (13). In this article we are providing an ethical analysis of the nature of vulnerability, and we are demonstrating that: (1) athletes are such a group that are “more than ordinarily vulnerable” and thus at risk of the harms of doping due to systemic pressures. We are also arguing that: (2) as a result of this special status, these individuals (athletes) require interventions, which we argue need to be (3) part of 'safeguarding' policies and practices, in order to alleviate their heightened vulnerability, and that those who are less vulnerable vis-a-vis athletes and who also are a part of the elite sport ecosystem have a moral responsibility to implement these interventions (20). By contrast, those who subscribe to the absolute or ontological notion of vulnerability, maintain that there are no sound grounds to affirm that emphasizing vulnerability as a universal ontological condition dilutes the normative significance of the concept, but rather, that acknowledging a universally shared vulnerability, presupposes a universally shared moral responsibility which can be traced back to the universal principle of no harm (21, 22) (see for example Butler's argument below).

Judith Butler, one of the more prominent post-modern feminist philosophers, suggests that “community” could be reimagined “on the shared basis of vulnerability and loss,” (15, 16) and that this reimagining could set the grounds for a communal ethical responsibility theory. We are proposing that in sports, athletes form a community where shared vulnerability, such as exposure to doping pressures and regulatory oversight, can be leveraged to build mutual support systems.

Whether relational or ontological, the concept of varying levels of vulnerability among humans raises important questions about the sources and implications of increased vulnerability, such as the moral obligations it entails. These questions are crucial for analyzing the normative significance of vulnerability, as increased vulnerability, such as that faced by athletes coerced into doping or unfairly sanctioned, is seen as inequitable, posing significant challenges for ethics and vulnerability theory. Although humans share the same inherent vulnerability due to their embodied, social, and political nature, the degree of susceptibility to harm varies among individuals (24). This increased potential for harm can stem from intrinsic qualities or specific circumstances, making some individuals, such as young athletes, athletes with limited resources, or those subject to intense training regimes and scrutiny, more vulnerable than others.

Acknowledging universal human vulnerability, as suggested by Butler and Miller, implies a focus on justice, as categorizing specific groups as vulnerable, like athletes under anti-doping scrutiny, indicates an inequitable distribution of this inherent trait. The moral inquiry should then focus on identifying the circumstances under which harm potential increases and individuals become more vulnerable, such as the pressures that lead athletes toward doping or the circumstances surrounding unfair sanctioning, rather than just labeling certain individuals as inherently more vulnerable or as moral failures.

Since vulnerability is understood as being at an increased risk of harm, vulnerability is closely linked to justice and autonomy. In sports, if an athlete's autonomy is hindered, such as through coercion to use performance-enhancing drugs, or through the lack of access to justice, they are at increased risk of harm, both physically and ethically, due to being less able to pursue their conception of the good and of a flourishing life freely and being more likely to be exploited by others. Threats to autonomy, particularly those associated with minor athletes, and athletes suffering from mental health conditions, can be a source of increased vulnerability, such as inherent negative influences on autonomy present with some mental health conditions or negative social influences that erode self-trust, such as the stigma of mental illness (e.g., Simone Biles). This stigma can also lead to athletes making desperate choices, including resorting to banned substances, to meet expectations. It is important to note, though, that not all sources of vulnerability are due to injustice, but some can be. However, systemic sources of vulnerability—like the lack of support systems for athletes to resist doping pressure or to access justice avenues, or other sources of vulnerability that could reasonably be reduced but are not — could be an injustice. Moreover, building on the moral responsibilities that stem from increased vulnerability, systemic sources of vulnerability could also be a failure of sport authorities' obligations to meet their duty of care in ensuring athletes' right to safe sport^1^ (26).

This approach shifts at least part of the responsibility for increased vulnerability from individuals to the contexts or circumstances, suggesting that rectifying these external factors can mitigate harm. For instance, examining the Olympic team selection decision-making process reveals how systemic pressures can contribute to an athlete's choice to dope, as these pressures often influence the vulnerabilities athletes experience. Addressing these factors could reduce the instances of doping by altering the context that exacerbates an athlete's vulnerability. A key ethical question related to the concept of vulnerability is whether we are morally required not only to avoid harming others e.g., as argued by Mill (27) and Feinberg (28) but also to actively prevent harm and assist those who are suffering, e.g., as argued by Hume (29) which in turn, could be directly related to safeguarding commitments. Conceptual clarity on vulnerability can illuminate who the vulnerable are, identify the specific harms a group is susceptible to, and help to establish a moral response that addresses increased susceptibility to harm. In sports, this involves identifying, for example, not only athletes who are particularly at risk of resorting to doping and formulating strategies to protect them from the pressures that drive them toward such actions, but also other harms present within the world anti-doping program, such as the mentioned unjust sanctions, or the difficulties in accessing justice. It also helps clarify who has a duty to protect the vulnerable, such as sporting organizations, coaches, and policymakers, and what those duties entail in terms of minimizing the risks associated with doping.

In this sense described above and building on Mackenzie et al. *(*11), an ethics of vulnerability must begin by addressing four fundamental questions: (i) What is vulnerability and its different types and which ones apply to elite athletes? (ii) Why vulnerability give rise to moral obligations and duties of justice in the context of sport? (iii) Who bears primary responsibility for responding to athlete vulnerability? And (iv) how are our obligations to the vulnerable athletes best fulfilled?

## 5 Definitions of safeguarding

Safeguarding in the English language is defined as keeping secure from danger or attack; to guard, protect, defend; to make safe (30). Historically, in public policy, there are examples of safeguarding from child welfare in the UK (early 2000s) associated with protection and flourishing for all British children (31). In the context of sport, research in the realm of psychology and sociology has occurred in response to harassment and abuse (26, 32, 33). Some areas of research include identifying individual behaviors impacting athletes' integrity and well-being, establishing direct correlations between conducts and detrimental effects, and seeking prevalence (26). Gurgis and Kerr defined sport safeguarding as “the prevention of harm and the promotion of positive values in sport” (33), and the latest Consensus Statement on Interpersonal Violence and Safeguarding in Sport issued by the International Olympic Committee (IOC) defines safeguarding as “All proactive measures to both prevent and appropriately respond to concerns related to harassment and abuse in sport as well as the promotion of holistic approaches to athlete welfare.” (34). The latest consensus statement also offers a recently updated definition of Safe Sport understood as “A physically and psychologically safe and supportive athletic environment where participants can thrive and experience the full benefits of sport participation.”. The earlier definition of Safe Sport was also coined by the IOC, in its earlier consensus statement on Harassment and Abuse (non-accidental violence) in Sport as “an environment that is respectful, equitable, and free from all forms of harassment and abuse” (26). The research work that has been compiled by these consensus statements are the reference framework for safeguarding policy worldwide, and has led the focus of current state-of-the-art safeguarding policies, built primarily, up until 2024, before the latest statement, upon lists of proscriptions i.e., “Don’t do this..” “these conducts (e.g., harassment and abuse) are prohibited.”

'Safeguarding' also covers the protection of health, well-being and human rights, and effective safeguarding enables people (particularly children, young adults and other vulnerable people) to thrive and live free from fear of abuse, harm or neglect (35). A significant focus of current safeguarding policies includes several forms of interpersonal violence such as psychological abuse, physical abuse, sexual abuse, and neglect, and approaches safeguarding as an active verb (34). An ethics-based approach to safeguarding is proactive in keeping all those at risk safe from harm or abuse by ensuring the well-being, dignity, and rights of individuals, particularly those who may be vulnerable to exploitation (such as minors or adults with disabilities). This approach aims to actively promote their autonomy, agency, and flourishing.

## 6 Vulnerability in the anti-doping context

While it is undeniable that values and moral reasoning play a crucial role in shaping athlete's attitudes towards anti-doping and influencing their decisions regarding whether or not to use performance-enhancing substances and methods, current literature has predominantly emphasized these individual moral considerations. This focus has led to a relative neglect of the broader structural and situational factors that contribute to athletes' susceptibility to doping, specifically their experiences of vulnerability and their exposure to it. Thus, a more comprehensive framework that integrates both individual moral agency and the vulnerabilities that may predispose athletes to engage in doping practices is needed. This approach would not only enrich our understanding of doping behaviors but also provide a more effective basis for developing prevention strategies and safeguarding policies that address the root causes of doping beyond mere moral failure, e.g., Aristotle's *Akrasia* or “weakness of will” when a competent person acts freely and intentionally against their better judgment (36).

Based in part on Veltmaat et al.'s (6) empirical study, we will develop a theoretical analysis of vulnerabilities, doping and safeguarding. Their research explores the role of contextual factors such as sporting environments, personal traits and educational access in shaping athletes' vulnerability to doping. These insights and others outlined below (Overview of Psychologically based research on vulnerability and anti-doping**)** form part of the basis for justifying a *safeguarding approach*, which is one of the primary points of this paper, that addresses both moral and contextual dimensions in the fight against doping. In this article, we have further explored the concept of vulnerability, and its relationship with doping risks, through an intersectional lens drawing on biomedical ethics and feminist critical analysis. Our examination of this relationship between vulnerability and the anti-doping context has been intended to provide a more developed understanding of anti-doping as a matter of safeguarding and a moral obligation for sport governing bodies and anti-doping organizations to protect athletes from harm and reduce vulnerabilities.

## 6.1 Overview of psychologically based research on vulnerability and anti-doping

Although this paper focuses primarily on the ethical and conceptual relationship between safeguarding and vulnerability in the anti-doping context, it is equally important to understand how vulnerability has been explored in empirical psychological research. This body of work, from foundational studies such as Petróczi and Aidman (19), through empirical work on susceptibility (37, 38), to more recent work expanding vulnerability to clean athletes (39, 40), has been instrumental in identifying how structural, interpersonal, and psychological pressures affect athletes' decisions regarding performance-enhancing substances. These insights help ground our normative arguments in lived realities and highlight the practical urgency of a safeguarding approach.

Petróczi and Aidman's life-cycle model remains a key contribution, offering a view of vulnerability across individual (e.g., personality traits, self-esteem), systemic (e.g., motivational climate, authority structures), and situational (e.g., peer pressure, access to substances) dimensions (19). Their work demonstrates how susceptibility to doping evolves across an athlete's career and how the progression from legal to illegal enhancement practices is often shaped by environmental and relational factors, not simply moral failings. This model provides strong empirical support for moving beyond a punitive, deterrence-based anti-doping framework and instead addressing the underlying pressures that make athletes vulnerable to harm.

Building on this, Martinelli et al. explore the experiences of self-identified clean athletes, showing that they too face psychological stress and institutional pressure under current anti-doping regimes (39). Their concept of “clean anxiety” captures the fear of inadvertent rule violations and reputational damage, even among those committed to drug-free sport. This stress is compounded by a lack of institutional support and transparency, which undermines athletes' trust in the system and contributes to a broader sense of vulnerability shaped by power imbalances and uncertainty.

Piffaretti et al. offer further insight into how anti-doping rule violations can lead to cascading social, emotional, financial, and psychological consequences, including significant mental health challenges (40). Their study advocates for a shift toward education, rehabilitation, and support—framing doping not merely as a moral transgression, but as a health and welfare issue often exacerbated by systemic conditions. They identify doping as a form of institutional harm, particularly when sanctions are imposed without adequate support or understanding of the pressures that led to the violation.

Van der Kallen et al. extend this discussion by analyzing the long-term biopsychosocial effects of doping bans, including career disruption, mental health deterioration, and social isolation (41). Notably, they emphasize the ethical responsibility of sports organizations to continue safeguarding athletes' well-being even after sanctions are imposed. Their attention to unintentional doping, now accounting for a significant proportion of cases, reinforces the view that vulnerability often stems from structural and environmental contexts rather than individual intent alone.

Veltmaat et al. introduce a model that frames doping vulnerability as a dynamic balance between risk factors (e.g., external pressures, temptation, systemic enablers) and resilience factors (e.g., coping strategies, values, and education) (6). Their findings stress that athletes' decisions are shaped by far more than personal values or moral reasoning; they are embedded in social environments that can either reinforce or weaken ethical behavior. Concepts such as moral disengagement and normalization of doping behaviors further highlight how culture and context influence vulnerability (42).

Taken together, this body of research illustrates that doping vulnerability is not simply an individual issue but a systemic one, relationally constructed through social, institutional, and psychological factors. These studies offer empirical validation for our theoretical framework grounded in feminist bioethics and theories of relational vulnerability. They also highlight the pressing need to develop athlete-centered policies that not only educate and deter but also support and safeguard, especially in moments of heightened risk. Psychological research thus plays a crucial role in strengthening the ethical case for safeguarding in sport and underscores the importance of shifting anti-doping policy from a model of punishment to one of care, prevention, and structural accountability.

## 7 Vulnerability, safeguarding and minors in the anti-doping context

Over the past two decades, research has shown that doping is not limited to adult athletes; young individuals have also been found to use performance-enhancing substances and methods. Studies indicate that the prevalence of doping among adolescent athletes may range from 3% to 12% (43–45), which translates to a significant number of young athletes potentially engaging in doping practices. This phenomenon raises serious concerns about the ethical implications and long-term health consequences of doping in youth sports.

Doping cases in young athletes exemplify the complexities surrounding specific vulnerabilities and doping among adolescents in sport. For instance, Rick De Mont, an American swimmer, became a prominent figure in the doping debate when he was disqualified from the 1972 Olympics due to a positive test for a banned substance, which he claimed was an asthma medication. While De Mont was an adult at the time, the incident set a precedent that ripples through the realm of sports, affecting perceptions of doping across all age groups. Similarly, Andreea Raducan, a young Romanian gymnast, faced a similar fate at the 2000 Sydney Olympics when she tested positive for a banned substance after taking a cold medication. At just 16 years old, Raducan became a symbol of the tragic consequences facing young athletes, highlighting the often-troubled realities of drug use and the potential for unintentional doping. The emotional toll of being labeled a cheater at such a young age can have profound repercussions on an athlete's mental health and sporting future. One of the most troubling cases is that of Geneviève Jeanson, a Canadian cyclist who revealed that her positive doping control was a desperate attempt to escape an abusive context within her sport. Jeanson's story underscores the multifaceted nature of doping, intertwining issues of personal safety, mental health, and systemic abuse in competitive sports. Her narrative sheds light on how young athletes may feel pressured to conform to unhealthy practices not just for performance enhancement but as a coping mechanism in toxic environments. It also speaks strongly to the need for safeguarding to be addressed in the anti-doping context.

The experiences of these young athletes underscore the pressing need for a comprehensive *safeguarding approach* to doping prevention that takes into account the unique vulnerabilities of youth in sports. Factors such as peer pressure, the desire for success, and the often-abusive nature of competitive environments contribute significantly to adolescents' susceptibility to engage in doping practices. Moreover, the increasing commercialization of youth sports has created a culture where winning is prioritized over well-being (46). Young athletes, often aspiring to achieve professional status, may feel immense pressure to perform at levels that are unsustainable without assistance from performance-enhancing drugs.

In turn, the inclusion of the concept of “protected persons” in the WADA Code speaks of the rising concern about athlete safeguarding and pediatric doping from a policy perspective, but also about recognition of athletes’ vulnerabilities. WADA considers protected persons to be those under the age of 16 or 18 under certain conditions (47). In a comprehensive examination of the WADA “Protected Person” category, Campos, Parry, and Martinkova (48) critically assess the shortcomings of this concept, both in terms of normative principles and conceptual framework. Relevant aspects of this critique resonate with our position, as we consider that the concept of “protected persons” requires a deeper philosophical debate that would, over time, be reflected in the design of policies to adequately safeguard vulnerable athletes from doping and the potential impact of anti-doping policies and protocols. Concepts such as vulnerability and safeguarding must be central to a common understanding of a protected person in the framework of the World Anti-doping Program.

As we have articulated, although values are central to doping behaviors, they are not the only aspect to take into account. Contextual vulnerabilities also matter. A focus on moral failure intensifies the threats to the athlete's well-being. Doping is not yet clearly perceived as a form of abuse in sport, as much attention is placed on athletes' ethical shortcomings. Therefore, at present, safeguarding approach responses to protect vulnerable athletes against doping are scarce. Our proposal to build upon Mackenzie's kind of ethical framework regarding vulnerability, adopts a transcendent perspective to address the prevalent, overly pessimistic perception of vulnerability within ethics in sport.

At first glance, it might appear that vulnerability and autonomy stand in conflict with one another. However, embracing a transcendent approach allows us to deal with this apparent dichotomy. Autonomy, defined as both the capacity to lead a self-directed life and the recognition of one's status as an autonomous individual by society, plays a crucial role in achieving a fulfilling existence (49). This is especially pertinent in the context of doping; for instance, athletes like Genevieve Jeanson have been pressured into using performance-enhancing drugs (PEDs) due to external expectations, leading to a loss of personal autonomy in decision-making. Therefore, it would be a fundamental error for an ethical framework centered on vulnerability to dismiss the concept of autonomy or its significance in the quest for equality.

In the discourse surrounding sports ethics, the perspectives of John Russell (50) and Nicolas Dixon (51) resonate with this viewpoint. In examining children's participation in risky sports, Russell asserts that while we do not condone the acceptance of risks associated with the use of PEDs by young athletes, we recognize that paternalistic approaches often fail to promote genuine autonomy. For example, in the case of young athletes who are coerced into using steroids to enhance performance to meet expectations from coaches or peers, paternalism may exacerbate vulnerabilities rather than alleviate them. Russell remarks, “The key element is clearly the determination that the person for whom we are acting is in fact not acting voluntarily, perhaps due to immaturity, ignorance, incapacity, or coercion…There is a place for paternalistic interference when beneficiaries are immature or incompetent.” (50) This highlights the tension that arises when individuals are not adequately equipped to make informed choices about their bodies and careers. In such contexts, paternalism can sometimes reinforce the same power dynamics that contribute to athletes' vulnerabilities. By focusing on preventing harm through restrictions, paternalistic interventions may inadvertently strip athletes, particularly younger ones, of their agency and reinforce the pressures from external forces such as coaches or institutions. The challenge lies in balancing protective measures with the empowerment of athletes to make choices about their own well-being, thus promoting genuine autonomy rather than mere compliance with paternalistic regulations. This balance is crucial in addressing the ethical complexities of doping and safeguarding in sports.

Furthermore, the WADA Code (52)treats “protected persons” differently from other athletes based on the understanding that individuals below a certain age or intellectual capacity may lack the mental maturity necessary to fully comprehend and appreciate the prohibitions against certain conduct established by the Code. A compelling example of the consequences young athletes can suffer from sanctions is the case of Andreea Raducan, who was stripped of her Olympic gold medal after testing positive for a banned substance at a young age. Despite its lack of intent, the severity of the punishment highlights why the Code's protections are essential to safeguarding young athletes. This differentiation underscores the need to protect the welfare of younger athletes, who may be particularly vulnerable to coercion, negligence or manipulation by adults in sport. It makes a strong case for stronger safeguards to prevent such outcomes.

Despite the recognition by moral theorists, philosophers, biomedical ethicists, and policymakers of the normative significance of human vulnerability, systematic analyses of the concept have been limited. The challenges associated with effectively addressing vulnerability in various contexts, including sports, often stem from a foundational issue: a lack of comprehensive understanding of what vulnerability truly entails. For instance, the vulnerability of athletes facing doping allegations—such as the public scrutiny experienced by Rick De Mont—demonstrates the need for a deeper exploration of the circumstances that contribute to these vulnerabilities. It is therefore essential to delve deeper into the nature of vulnerability in the context of anti-doping in sport.

Traditionally, sport ethics have understood vulnerability in terms of a lack of capacity or inherent weakness, often following Kantian frameworks of autonomy that emphasize individual agency (52, 53) However, this understanding, which is also reflected in the dominant approach of anti-doping organizations and sport governing bodies, places vulnerability in direct opposition to autonomy. The prevailing notion suggests that the freedom of decision-making must be balanced with the imperative to protect individuals from making detrimental choices.

As we have discussed, vulnerability is frequently framed within relational theories of autonomy, characterized by an increased risk of harm and/or a diminished ability to protect oneself from such harm, particularly in cases of athletes under pressure to use PEDs to compete successfully. However, a stringent focus on assessments of capacity is insufficient, as it fails to address the underlying sources of vulnerability. For instance, when examining the systemic pressures within competitive environments, such as the culture of doping in professional cycling, we find that many athletes feel compelled to engage in drug use to remain competitive (54). Our preceding analysis leads to the requirement of the adoption of a broader conception of vulnerability, one that integrates concepts from relational vulnerability. This expanded framework acknowledges that the traditional liberal conception of autonomy is overly individualistic and neglects the social and relational factors that influence an individual's circumstances. Capturing the social influences on vulnerability is essential for comprehensively understanding the complexities involved in the ethical discourse surrounding doping, particularly for young athletes within the realm of sport.

## 8 Discussion and conclusions

In summary, we have provided an ethical analysis of the nature of vulnerability and safeguarding and demonstrated that: (1) athletes are “more than ordinarily vulnerable” and thus at risk of the harms of doping due to systemic pressures and also other harms, such as unjust sanctions or the difficulties in accessing justice; (2) as a result of this special status, these individuals (athletes) require interventions, which need to be (3) part of 'safeguarding' policies and practices, in order to alleviate their heightened vulnerability, and that those who are less vulnerable vis-a-vis athletes and who also are a part of the elite sport ecosystem have a moral responsibility to implement these interventions.

By reconceptualizing vulnerability through a relational lens, and connecting it clearly to safeguarding, we can foster a more nuanced understanding that not only respects the autonomy of athletes but also recognizes the contextual factors that contribute to their vulnerabilities. This dual recognition is vital for developing ethical frameworks that promote both autonomy and protection, ensuring that all athletes—whether they are dealing with the temptation to use PEDs or facing the repercussions of doping allegations—can navigate their sporting endeavours with informed agency.

Educational programs aimed at raising awareness about the risks of performance-enhancing drugs are essential. However, these programs should emphasize not only the health risks associated with doping, but also the ethical considerations of fair play and integrity in sports. Psychological support systems are equally important, providing young athletes with coping strategies to handle competitive pressures and the emotional challenges of athletic life. In addition to education, creating supportive environments that prioritize athletes' health and well-being can significantly reduce the temptation to dope. Coaches, parents, and sports organizations must work together to foster a safe culture of integrity, emphasizing that the value of sports lies not solely in winning but in personal growth, resilience, and camaraderie.

By infusing elements of feminist theory into the examination of vulnerability as a concept and demonstrating its powerful relationship with safeguarding, we can uncover alternative perspectives, notably a profoundly relational interpretation of vulnerability that challenges the misleading dichotomy with autonomy and overcomes negative connotations of vulnerability in sport. A pressing requirement exists for the development of a conceptual and ethical framework pertaining to vulnerability and safeguarding within the domain of sports and anti-doping. Such a framework should not only strive for conceptual precision but also underscore its normative significance. Consequently, it can elucidate the question of which parties should assume the responsibility for addressing various forms of vulnerability and safeguarding and how to most effectively discharge this duty while concurrently fostering autonomy.

We recommend future research that can advance: (i) knowledge of vulnerable athletes' experiences of the impact of safeguarding issues specifically in the anti-doping context; and (ii) knowledge mobilization efforts through the development of strategies for additional capacity for the WADA to supplement and adapt current resources and disseminate them to athletes, sports organizations and teams.

Moreover, engaging coaches, parents, and sports organizations in this educational initiative can create a supportive environment where athletes feel safe discussing their concerns and seeking guidance. By cultivating a culture of integrity and transparency, we can mitigate the factors that contribute to doping vulnerabilities, ultimately fostering healthier sporting environments that prioritize athlete well-being. This preventative framework should also include regular monitoring and support systems that allow athletes to voice their struggles and seek help without fear of stigma or retribution—an essential aspect of safeguarding. Rather than relying solely on a punitive model, the focus should shift toward education and support, ensuring that athletes are equipped to make informed choices and uphold the integrity of their sport in a safe environment.

## Data Availability

The raw data supporting the conclusions of this article will be made available by the authors, without undue reservation.

## Footnotes

^1^Where safe sport was defined as “An athletic environment that is respectful, equitable, and free from all forms of non-accidental violence” (25)
